# Waves in elastic bodies with discrete and continuous dynamic microstructure

**DOI:** 10.1098/rsta.2019.0313

**Published:** 2019-11-25

**Authors:** Gennady S. Mishuris, Alexander B. Movchan, Leonid I. Slepyan

**Affiliations:** 1 Department of Mathematics, IMPACS, Aberystwyth University, Aberystwyth SY23 3BZ, UK; 2 Department of Mathematical Sciences, University of Liverpool, Liverpool L69 7ZL, UK; 3 School of Mechanical Engineering, Faculty of Engineering, Tel Aviv University, PO Box 39040, Ramat Aviv, 69978 Tel Aviv, Israel

**Keywords:** dynamic microstructure, wave equations, Green's functions, integral transforms, dispersion relations

## Abstract

This paper presents a unified approach to the modelling of elastic solids with embedded dynamic microstructures. General dependences are derived based on Green's kernel formulations. Specifically, we consider systems consisting of a *master* structure and continuously or discretely distributed oscillators. Several classes of connections between oscillators are studied. We examine how the microstructure affects the dispersion relations and determine the energy distribution between the master structure and microstructures, including the vibration shield phenomenon. Special attention is given to the comparative analysis of discrete and continuous distributions of the oscillators, and to the effects of non-locality and trapped vibrations.

This article is part of the theme issue ‘Modelling of dynamic phenomena and localization in structured media (part 2)’.

## 1. Introduction

In contrast to solid-state physics, where crystalline solids and periodically structured media are considered as a standard framework, the preference in solid mechanics is given to continuous models. Even the theories of elastic composites are dominated by homogenization approaches, which are convenient in the calculations widely used in the theories of material strength. Such a continuous model is flawless as long as micro-scale oscillations or waves are not excited. It can, however, occur in the case of moving singular points (as, for example, in fracture) or in metamaterials where the micro-scale dynamics play the crucial role.

### (a) Overview

In dynamic elasticity, the fundamental ideas of modelling solids as a structured medium were developed many decades ago. This includes the classical nineteenth-century work by Navier and Poisson, discussed in the articles by Arnold [1] and Todhunter [2], and contributions by Maradudin *et al.* [3], Novozhilov [4] and Kunin [5,6]. The micromechanics of materials is comprehensively discussed in the book by Kachanov & Sevostianov [7].

The role of microstructure was emphasized in scattering problems associated with systems possessing many defects (scatterers) or in waves by Karp & Karal [8], Hills [9], Hills & Karp [10] and Slepyan [11,12]. Kunin [5,6] made a fundamental contribution to the theory of elastic media with microstructure. In particular, Green's functions (or Green's tensors) are essential in understanding the physical fields around defects. The periodic and quasi-periodic Green's functions have a special role in the modelling of microstructured solids and the analysis of waves in periodic composites [13].

Microstructure plays a crucial role in fracture mechanics. Griffith [14] formulated a fracture criterion based on surface energy. The latter represents the characteristic length scale and an implicit homogenization for a solid with microstructure. Furthermore, the work of Novozhilov [15,16] made a significant advance by emphasizing discreteness and instability as primary factors associated with the fracture phenomenon. In the dynamic response, the effect of microstructure becomes even more apparent, as it explains energy dissipation via waves propagating away from the crack tip.

Lattice trapping is found in quasi-static crack growth by Thomson *et al.* [17] and in dynamic crack propagation (where it is dependent on crack speed) by Slepyan [18–21] and Kulakhmetova *et al.* [22]. Dispersion of waves in the structured medium is also linked to dynamic crack stability in a structured solid, as explained in detail in Marder & Gross [23]. Some non-trivial phenomena in lattice dynamics and fracture have been revealed: a binary crack in a triangular lattice [24], and primitive waveforms and star waves in lattices [25], and also in non-homogeneous and bridged cracks [26–28]. Non-steady clustering crack modes were discovered by Mishuris *et al.* [29,30]. The admissibility conditions introduced by Marder & Gross [23] proved to be necessary for steady-state crack propagation analysis.

Note that energy trapping by elastic waves in discrete waveguides, homogenization approximations and their limitations were also discussed by Evans & Porter [31], Linton & Martin [32] and Haslinger *et al.* [33,34], and recently for a finite circular cluster of resonators connected to an elastic solid by Movchan *et al.* [35].

The proposed framework is linked to the Slepyan [11] model of a homogeneous elastic cylinder connected to a continuous mass–spring shell, where a longitudinal elastic wave with an expanding quasi-front was examined. The added oscillators were assumed to be uniformly distributed over the cylinder and arbitrarily distributed in frequency.

The microstructured systems considered here belong to the class often referred to as ‘metamaterials’. This term is linked to work by John Pendry and Victor Veselago (see [36]). The idea of geometric transformations in the design of new materials was proposed by Dolin [37]. Among several interesting features of metamaterials, waves may show unusal dispersion properties, negative refraction and negative inertia. In particular, a three-dimensional elastic medium with a microstructure, which allows for negative inertia, is discussed in detail by Milton & Willis [38].

### (b) Plan of the paper

We propose a general theoretical framework for controlling waves in dynamic multi-structures, which incorporate connected continuous or discrete resonators. Four types of networks with different interconnections between resonators and the master structure are discussed in §3b.

The structure of the paper is as follows. First, we give a generic theoretical framework for three-dimensional dynamic elasticity, with a microstructure modelled via non-local operators of the convolution type. A spherical wave propagating in a three-dimensional elastic body with such microstructure is discussed as an example.

We then demonstrate various physical features of such systems by presenting and analysing in detail applications of the general theory to a string and to a flexural elastic beam with the mass–spring structure attached. We consider discretely and continuously distributed oscillators, connected or otherwise to their nearest neighbours. We also consider homogenization approximations and establish conditions under which the effective continuous distribution is justified. In this way, we use the continuous and discrete Fourier transforms as a unifying mathematical tool (see §2.5 in [39]).

**Figure 1.**
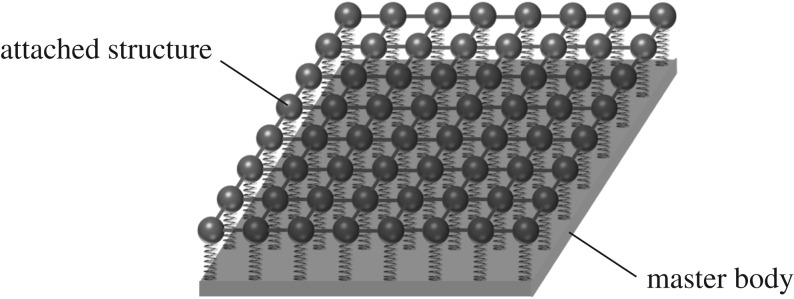
Example of a two-dimensional continuous flexural plate and discretely distributed interconnected resonators.

In the entire dynamic multi-structure, we identify the ‘master system’ as well as an additional dynamic system of resonators as in figure 1. The main effects of interaction between these components of the dynamic multi-structure are (i) the appearance of stop bands in the dispersion diagrams, (ii) a remarkable energy partition between the two structures (a significant part of the wave energy can settle on the microstructure) and (iii) a reduction of the group velocity in the combined system, which results in wave localization. The localized waves are predicted and observed in certain frequency regimes, as also discussed in Evans & Porter [40,41], Haslinger *et al.* [33,34] and Movchan *et al.* [35].

For waves in a system with continuously and discretely distributed oscillators, we evaluate the ratio of wave amplitudes in the master structure and in the embedded substructure. It appears that the amplitude in the master system tends to zero as the wave frequency approaches the band gap boundary. We discuss this fascinating phenomenon in detail.

In particular, we found that the microstructure considered lowers the wave velocity, which, in turn, results in its localization. We present an analysis of this phenomenon in detail separately.

## 2. Three-dimensional elastic medium with embedded microstructure

### (a) Derivation of the dynamic equations

The Navier–Lamé dynamic equation for a homogeneous, isotropic, linearly elastic medium is

2.1 $$\mu \Delta u+(\lambda +\mu )\nabla (\nabla \cdot u)-\varrho \frac{\partial^{2}u}{\partial t^{2}}=-p,$$

where ***u*** and ***p*** are the displacements and a body force, respectively. We represent the latter as the sum of an external body force ***p***_1_ acting directly on the medium and a force exerted by the embedded microstructure.

In turn, the latter is defined by its dynamics under the displacement ***u*** and the external force ***p***_2_ acting on the microstructure directly:

2.2 $$p=p_{1}-R**\;u+M**\;p_{2},$$

where R and M are some convolution operators characterizing the microstructure. Assuming its mechanical properties are independent of space and time,

2.3 $$\left.\begin{array}{rl} R(x,t)**\;u(x,t) & =\int_{-\infty }^{\infty }\int_{-\infty }^{\infty }u(\xi ,\tau )R(x-\xi ,t-\tau )\;d\xi \;d\tau \\ \mathrm{and}\;\;M(x,t)**\;p_{2}(x,t) & =\int_{-\infty }^{\infty }\int_{-\infty }^{\infty }p_{2}(\xi ,\tau )M(x-\xi ,t-\tau )\;d\xi \;d\tau , \end{array}\right\}$$

then, in these terms, equation (2.1) becomes

2.4 $$\mu \Delta u+(\lambda +\mu )\nabla (\nabla \cdot u)-\left(\varrho \frac{\partial^{2}}{\partial t^{2}}+R**\;\right)u=-p_{1}-M**\;p_{2},$$

where R can be considered as added non-local inertia. It plays a crucial role in understanding of the role of the microstructure in wave propagation and dispersion.

### (b) General formulation in terms of the dynamic potentials

We represent the vector fields ***u*** and ***p***_1,2_ via scalar and vector potentials (as any vector fields can be expressed)

2.5 $$\left.\begin{array}{rl} u & =\nabla \phi +\nabla \times \psi \\ \mathrm{and}\;p_{1,2} & =\nabla \Phi_{1,2}+\nabla \times \Psi_{1,2}. \end{array}\right\}$$

Substitution of these expressions into (2.4) leads to

2.6 $$\begin{array}{rl} & \nabla \left[(\lambda +2\mu )\Delta \phi -\left(\varrho \frac{\partial^{2}}{\partial t^{2}}+R**\;\right)\phi +\Phi_{1}+M**\;\Phi_{2}\right]\\ & \;+\nabla \times \left[\mu \Delta \psi -\left(\varrho \frac{\partial^{2}}{\partial t^{2}}+R**\;\right)\psi +\Psi_{1}+M**\;\Psi_{2}\right]=0. \end{array}$$

Thus, taking the microstructure into account, the Sobolev wave equations [42] become

2.7 $$\left.\begin{array}{rl} & (\lambda +2\mu )\Delta \phi -\left(\varrho \frac{\partial^{2}}{\partial t^{2}}+R**\;\right)\phi =-\Phi_{1}-M**\;\Phi_{2}\\ \mathrm{and}\;\; & \mu \Delta \psi -\left(\varrho \frac{\partial^{2}}{\partial t^{2}}+R**\;\right)\psi =-\Psi_{1}-M**\;\Psi_{2}. \end{array}\right\}$$

Under zero initial conditions, the LF transform (the Laplace transform in time *t* and the Fourier transform in spatial variable ***x***) leads to

2.8 $$\left.\begin{array}{rl} & (\lambda +2\mu )k^{2}\phi^{\mathrm{LF}}-(\varrho s^{2}+R^{\mathrm{LF}})\phi^{\mathrm{LF}}=-\Phi_{1}^{\mathrm{LF}}-M^{\mathrm{LF}}\Phi_{2}^{\mathrm{LF}}\\ \mathrm{and}\; & \mu k^{2}\psi^{\mathrm{LF}}-(\varrho s^{2}+R^{\mathrm{LF}})\psi^{\mathrm{LF}}=-\Psi_{1}^{\mathrm{LF}}-M^{\mathrm{LF}}\Psi_{2}^{\mathrm{LF}}. \end{array}\right\}$$

In particular, if the tensor operator R is isotropic and acts locally in space,

2.9 $$R=R_{0}\prod_{i=1}^{3}\delta (x_{i}),$$

where $R_{0}$ is a time operator, then the convolution on the space coordinates is an identity tensor and only the time convolution remains.

If the embedded structure consists of several substructures, the superposition principle implies that the combined action of different structures corresponds to the sum of related integral operators. Thus, equations (2.7) can be expressed in a more general way as

2.10 $$\left.\begin{array}{rl} & (\lambda +2\mu )\Delta \phi -\left(\varrho \frac{\partial^{2}}{\partial t^{2}}+\sum_{i}R_{i}**\;\right)\phi =-\Phi_{1}-\sum_{i}M_{i}**\;\Phi_{2i}\\ \mathrm{and}\; & \mu \Delta \psi -\left(\varrho \frac{\partial^{2}}{\partial t^{2}}+\sum_{i}R_{i}**\;\right)\psi =-\Psi_{1}-\sum_{i}M_{i}**\;\Psi_{2}, \end{array}\right\}$$

where the sums can also be replaced by integrals as in Slepyan [11], where the attached oscillators were continuously distributed in space and arbitrarily distributed in the frequency domain.

Note that the homogenization approach is not used here, and instead the embedded system of resonators is taken into account via the forcing term ***p*** in the equations of motion. Prior to the Green's function formulation, we discuss an example of a spherical wave in a medium with microstructure.

### (c) Spherical wave in a three-dimensional body with dynamic microstructure

Here we consider a symmetric spherical wave in a homogeneous isotropic structured composition, where the microstructure is represented by a micro-oscillating medium using homogeneously distributed oscillators. In this case, there are no external forces, ***p***_1_ = ***p***_2_ = 0, and also ***ψ*** = 0. The reciprocal Green's function −R follows from the equation

2.11 $$\varrho_{0}\ddot{v}=\varkappa (u-v)$$

in the oscillation medium, where ϱ_0_, ϰ and *v* are the density, the oscillator link stiffness and the displacement of the oscillator, respectively. Note that, in these terms, the oscillator frequency $\omega_{0}=\sqrt{\varkappa /\varrho_{0}}$.

It follows that the reactive stress caused by the displacement pulse *u* = *δ*(*x*_1_)*δ*(*x*_2_)*δ*(*x*_3_)*δ*(*t*) is (see (2.9))

2.12 $$-R_{0}=\varkappa \omega_{0}(-\delta (t)+\sin\omega t)H(t+0)\;\mathrm{and}\;R_{0}^{L}(s)=\frac{\varkappa s^{2}}{s^{2}-\omega_{0}^{2}}.$$

Equations (2.8) become

2.13 $$\left.\begin{array}{rl} & \frac{\;d^{2}r\;\phi^{L}(r,s)}{\;dr^{2}}-\alpha^{2}(s)r\phi^{L}(r,s)=0,\;\psi =0\\ \mathrm{with}\;\; & \alpha (s)=\frac{s}{c_{1}}\sqrt{1+\frac{\varrho_{0}\omega_{0}^{2}}{\varrho (s^{2}+\omega_{0}^{2})}},\;c_{1}=\sqrt{\frac{\lambda +2\mu }{\varrho }}. \end{array}\right\}$$

The potential *ϕ*^L^ follows as

2.14 $$\phi^{L}(r,s)=C(s)\frac{1}{r}\;e^{-\alpha (s)(r-r_{0})}\;(r\geq r_{0}),$$

and the stress *σ*_*rr*_ as

2.15 $$\sigma_{rr}(r,t)=\lambda \Delta \phi +2\mu \frac{\partial^{2}\phi }{\partial r^{2}}=(\lambda +2\mu )\frac{\partial^{2}\phi }{\partial r^{2}}+2\lambda \frac{1}{r}\frac{\partial \phi }{\partial r}.$$

Let the wave propagate from a spherical cavity of radius *r*_0_, where *σ*_*rr*_(*r*_0_) = *σ*. It follows that

2.16 $$C(s)=\frac{r_{0}^{3}\sigma^{L}(s)}{\left(\lambda +2\mu )r_{0}^{2}\alpha^{2}(s)+4\mu (1+r_{0}\alpha (s)\right)},$$

and the displacement is

2.17 $$u^{L}(r,s)=\frac{\;d\phi^{L}(r,s)}{\;dr}=-C(s)\frac{1}{r^{2}}(1+r\alpha (s))\;e^{-\alpha (s)(r-r_{0})}.$$

In particular, for a harmonic load $\sigma (t)=\sigma_{0}\exp(i\omega t)$ (*s* → 0 + i*ω*), the forced wave follows from (2.16) and (2.17) as

2.18 $$\left.\begin{array}{rl} u(r,t) & =-C(0+i\omega )R(r)\;e^{-\alpha (i\omega )(r-r_{0})+i\omega t},\;R(r)=\frac{1}{r^{2}}+\frac{1}{r}\alpha (i\omega )\\ \mathrm{and}\;\alpha (i\omega ) & =\frac{i\omega }{c_{1}}\sqrt{1+\frac{\varrho_{01}}{1-\Omega^{2}}},\;\varrho_{01}=\frac{\varrho_{0}}{\varrho },\;\Omega =\frac{\omega }{\omega_{0}}. \end{array}\right\}$$

Here we have introduced the non-dimensional values ϱ_01_ and *Ω*, which are used repeatedly below.

With reference to the quantity 0 + i*ω*, we note that the use of the Laplace transform over time allows us to consider transient forced waves assuming zero initial conditions. A solution for harmonically oscillating waves can be obtained from the transient one using the limiting relations and the causality principle (see [19], §§2.1.6 and 3.3.2, respectively). In particular, for the forced harmonic wave of frequency *ω* we have to substitute *s* → 0 + i*ω*:

2.19 uF=limuLF(s,k)=uLF(0+iω,k)(s→iω, Re s>0).

In doing so, we exclude energy flux from the infinity and provide uniqueness, which can otherwise be lost in some conversions.

We can see in (2.18) that there is a band gap in *ω* for

2.20 $$1<\Omega <\sqrt{1+\varrho_{01}},$$

where the wave amplitude exponentially decreases with distance *r* from the source as the function *R*(*r*). Such a band gap is a characteristic phenomenon introduced by the oscillating microstructure. Below, in §4, we discuss it in more detail.

## 3. Green's kernels in formulations of wave problems in structured solids

Recall that Green's functions (or Green's tensors) are fundamental in understanding the physical fields around defects, and, in particular, periodic and quasi-periodic Green's functions have a special role in the modelling of microstructured solids and the analysis of waves in periodic composites. Here we illustrate the theory in the case of one spatial dimension when the variable *x* is scalar.

The notation *G*(*x*, *t*) and *G*_0_(*x*, *t*) is used for Green's functions corresponding to the master body and the attached elastic microstructure, respectively. The general three-dimensional formulation was discussed in the text above; here we consider, for the sake of simplicity, the scalar formulations. Without loss of generality, the formal procedure also extends to higher dimensions.

Although several substructures can be attached to the master body, in the present section, we assume only one type of embedded microstructure (otherwise, the sum of the convolution terms occurs in (2.10)).

We assume here that the attached and master structures are connected by massless links.

### (a) General solution in terms of the Laplace and Fourier transforms

As above, we use the Laplace and Fourier transforms in time and space, respectively. The latter can be continuous or discrete, or continuous in one coordinate and discrete in the other. For the discrete transform, we use the notation ( · )^Fd^.

Let *u*(*x*, *t*) and *v*(*x*, *t*) be the displacements of the master and attached structures, respectively.

The following representations hold:

3.1 $$\left.\begin{array}{rl} u(x,t) & =G(x,t)**\;(p_{1}(x,t)+Q(x,t)),\;Q=\varkappa (v-u)\\ \mathrm{and}\;\;v(x,t) & =G_{0}(x,t)**\;(p_{2}(x,t)-Q(x,t)), \end{array}\right\}$$

where *Q* is the tensile force in the link connecting the systems, *p*_1,2_ are the external forces acting on the master body and the added one, respectively, ϰ is the link stiffness and * * means the convolution on time and space. Using the Laplace and Fourier transforms, we find from (3.1) that in terms of reciprocal Green's functions $G^{\mathrm{LF}}=1/G^{\mathrm{LF}}$ and $G_{0}^{\mathrm{LF}}=1/G_{0}^{\mathrm{LF}}$, we have

3.2 $$\left.\begin{array}{rl} u^{\mathrm{LF}} & =\frac{S_{2}p_{1}^{\mathrm{LF}}+\varkappa p_{2}^{\mathrm{LF}}}{S},\;v^{\mathrm{LF}}=\frac{S_{1}p_{2}^{\mathrm{LF}}+\varkappa p_{1}^{\mathrm{LF}}}{S},\;Q^{\mathrm{LF}}=\frac{\varkappa (G^{\mathrm{LF}}p_{2}-G_{0}^{\mathrm{LF}}p_{1})}{S}\\ \mathrm{and}\;\;S_{1} & =G^{\mathrm{LF}}+\varkappa ,\;S_{2}=G_{0}^{\mathrm{LF}}+\varkappa ,\;S=S_{1}S_{2}-\varkappa^{2}. \end{array}\right\}$$

Note that if both systems or one of them are discrete, the corresponding Fourier transform is also discrete. Equivalently, we can write

3.3 $$v^{\mathrm{LF}}(k,s)=\frac{\varkappa u^{\mathrm{LF}}(k,s)+p_{2}^{\mathrm{LF}}}{S_{2}}\;\mathrm{and}\;Q^{\mathrm{LF}}=\frac{\varkappa (p_{2}-G_{0}^{\mathrm{LF}}u^{\mathrm{LF}})}{S_{2}},$$

where the expression for *u*^LF^ presented in (3.2) can also be written as

3.4 $$u^{\mathrm{LF}}=\frac{p_{1}^{\mathrm{LF}}+M^{\mathrm{LF}}p_{2}^{\mathrm{LF}}}{G^{\mathrm{LF}}+R^{\mathrm{LF}}},\;R^{\mathrm{LF}}=\frac{\varkappa G_{0}^{\mathrm{LF}}}{G_{0}^{\mathrm{LF}}+\varkappa }\;\mathrm{and}\;M^{\mathrm{LF}}=\frac{\varkappa }{G_{0}^{\mathrm{LF}}+\varkappa }.$$

These relations are valid if both systems are continuous or both are discrete. Note that, assuming no load is applied to the structure (*p*_1_ = 0, *p*_2_ = 0), we obtain the dispersion relation

3.5 $$G^{\mathrm{LF}}+R^{\mathrm{LF}}=0.$$

In the case where the master body is continuous, whereas the attached structure is discrete, as shown for example in figure 1, we have

3.6 $$v^{\mathrm{LFd}}=G_{0}^{\mathrm{LFd}}(p_{2}^{\mathrm{LFd}}-Q^{\mathrm{LFd}})\;\mathrm{and}\;Q^{\mathrm{LFd}}=\varkappa (v^{\mathrm{LFd}}-u^{\mathrm{LFd}}),$$

where the discrete transform of the continuous function *u* is (with attached structure support *x* = *an*, *n* = 0, ± 1, …)

3.7 $$u^{\mathrm{LFd}}(x,t)=\sum_{n=-\infty }^{\infty }u^{L}(an,s)\;e^{ikan}.$$

It follows that

3.8 $$Q^{\mathrm{LFd}}=\frac{\varkappa p_{2}^{\mathrm{LFd}}-\varkappa G_{0}^{\mathrm{LFd}}u^{\mathrm{LFd}}}{S_{2}},$$

and equations (3.4) become

3.9 $$\left.\begin{array}{rl} G^{\mathrm{LF}}u^{\mathrm{LF}} & =p_{1}^{\mathrm{LF}}+h^{\mathrm{LFd}},\;h^{\mathrm{LFd}}=-R^{\mathrm{LFd}}u^{\mathrm{LFd}}+M^{\mathrm{LFd}}p_{2}^{\mathrm{LFd}}\\ \mathrm{with}\;\;R^{\mathrm{LFd}} & =\frac{\varkappa G_{0}^{\mathrm{LFd}}}{G_{0}^{\mathrm{LFd}}+\varkappa },\;M^{\mathrm{LFd}}=\frac{\varkappa }{G_{0}^{\mathrm{LFd}}+\varkappa }. \end{array}\right\}$$

Thus, we have coupled both the continuous and discrete Fourier transforms. A procedure for continuing with such a coupling is given in Slepyan [39]. Specifically, the combined expressions (3.9) can be reduced to a single discrete transform via the following fact. If a function *f*^F^(*k*) represents the product of the continuous *g*^F^(*k*) and the discrete *h*^Fd^(*k*) transforms,

3.10 $$f^{F}(k)=g^{F}(k)h^{\mathrm{Fd}}(k)\;\mathrm{and}\;h^{\mathrm{Fd}}\left(k+\frac{2\pi }{a}\right)=h^{\mathrm{Fd}}(k),$$

then the corresponding discrete transform of *f*(*an*) is

3.11 $$f^{\mathrm{Fd}}(k)=h^{\mathrm{Fd}}(k)\frac{1}{a}\sum_{n=-\infty }^{\infty }g^{F}\left(k+\frac{2\pi n}{a}\right).$$

From this and (3.9) it follows that

3.12 $$\begin{array}{rl} u^{\mathrm{LFd}} & =-(R^{\mathrm{LFd}}u^{\mathrm{LFd}}-M^{\mathrm{LFd}}p_{2}^{\mathrm{LFd}})\frac{1}{a}\sum_{n=-\infty }^{\infty }{\left(G^{\mathrm{LF}}\left(k+\frac{2\pi n}{a},s\right)\right)}^{-1}\\ & \;+\frac{1}{a}\sum_{n=-\infty }^{\infty }{\left(G^{\mathrm{LF}}\left(k+\frac{2\pi n}{a},s\right)\right)}^{-1}p_{1}^{\mathrm{LF}}\left(k+\frac{2\pi n}{a},s\right). \end{array}$$

The respective dispersion relation in this particular case takes the form

3.13 $$1+\frac{1}{a}R^{\mathrm{LFd}}\sum_{n=-\infty }^{\infty }{\left(G^{\mathrm{LF}}\left(k+\frac{2\pi n}{a},s\right)\right)}^{-1}=0.$$

### (b) One-dimensional dynamic microstructures with embedded resonators

**Figure 2.**
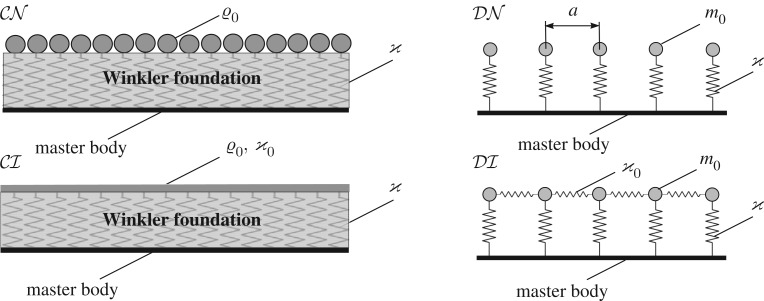
Four configurations of the oscillator system considered in this paper, attached to some master bodies: continuously distributed oscillators without interconnections (CN), continuously distributed locally interconnected oscillators (CI), discretely distributed oscillators not connected to each other (DN) and discretely distributed oscillators connected to each other (DI). Note that the stiffness ϰ has different dimensions in the continuous and discrete cases.

We consider four types of oscillating microstructure elastically connected to the master body (figure 2):

— continuously distributed oscillators not connected to each other but linked to the master body (case CN);
— continuously distributed oscillators with interconnections (case CI);
— discretely distributed not connected oscillators (case DN); and
— discretely distributed interconnected oscillators (case DI).

Note that the first case CN was considered in Slepyan [11] for an elastic rod equipped with distributed oscillators.

In the current paper, the master body is described by its transformed Green's function $G^{\mathrm{LF}}$ and in this section can be represented by a string. The combined multi-structures are shown in figure 2. The corresponding transformed Green's functions are as follows.

In the case CN we have

3.14 $$G_{0}^{\mathrm{LF}}=\varrho_{0}s^{2}\;\mathrm{and}\;R^{\mathrm{LF}}=\frac{\varrho_{0}\omega_{0}^{2}s^{2}}{s^{2}+\omega_{0}^{2}},$$

where the oscillator frequency $\omega_{0}=\sqrt{\varkappa /\varrho_{0}}$.

In the case CI we deduce

3.15 $$G_{0}^{\mathrm{LF}}=\varrho_{0}s^{2}+\varkappa_{0}k^{2}\;\mathrm{and}\;R^{\mathrm{LF}}=\frac{\varrho_{0}\omega_{0}^{2}(\varrho_{0}s^{2}+\varkappa_{0}k^{2})}{(s^{2}+\omega_{0}^{2})\varrho_{0}+\varkappa_{0}k^{2}}.$$

In the case DN, let the oscillators of mass *m*_0_ each be discretely set along the *x*-axis at *x* = *an*, *n* = 0,  ± 1, …; then

3.16 $$G_{0}^{\mathrm{LFd}}=m_{0}s^{2},\;R^{\mathrm{LFd}}=\frac{m_{0}\omega_{0}^{2}s^{2}}{s^{2}+\omega_{0}^{2}}\;\mathrm{and}\;\omega_{0}=\sqrt{\frac{\varkappa }{m_{0}}}.$$

Lastly, for the same system of oscillators linked to each other (the case DI) we have

3.17 $$G_{0}^{\mathrm{LFd}}=m_{0}s^{2}+2\varkappa_{0}(1-\cos ak)\;\mathrm{and}\;R^{\mathrm{LFd}}=\frac{m_{0}\omega_{0}^{2}(m_{0}s^{2}+2\varkappa_{0}(1-\cos ak))}{\left(s^{2}+\omega_{0}^{2})m_{0}+2\varkappa_{0}(1-\cos ak\right)}.$$

Note that in the cases CI and DI, the system represents by-pass waveguides.

In the following sections, we discuss dispersion relations corresponding to the above-mentioned four types of microstructure.

## 4. Dispersion of waves in a string equipped with continuously distributed oscillators

We begin with a simple example, where the master body is represented by an *elastic string*. In this case, $G^{\mathrm{LF}}=Tk^{2}+\varrho s^{2}$, where ϱ is the string mass per unit length, *T* is the tensile force, and equation (3.4) becomes

4.1 $$(Tk^{2}+\varrho s^{2}+R^{\mathrm{LF}})u^{\mathrm{LF}}=p_{1}^{\mathrm{LF}}+\frac{\varkappa p_{2}^{\mathrm{LF}}}{G_{0}^{\mathrm{LF}}+\varkappa }.$$

### (a) Continuously distributed oscillators without interconnections (case CN)

Referring to (3.14) we have the equation for a free complex wave exp⁡(i(ωt−kx)):

4.2 $$Tk^{2}-\omega^{2}\varrho \left(1+\frac{\varrho_{01}}{1-\Omega^{2}}\right)=0.$$

The *ω* - - *k* relationships are, in non-dimensional form,

4.3 $$K=\pm \Omega \sqrt{1+\frac{\varrho_{01}}{1-\Omega^{2}}}\;\text{or}\;\Omega =\pm \frac{\sqrt{2}}{2}\sqrt{1+\varrho_{01}+K^{2}\pm \sqrt{{\left(1+\varrho_{01}+K^{2}\right)}^{2}-4K^{2}}},$$

where

4.4 $$\Omega =\frac{\omega }{\omega_{0}},\;K=\frac{c_{1}k}{\omega_{0}},\;\varrho_{01}=\frac{\varrho_{0}}{\varrho }\;\mathrm{and}\;c_{1}=\sqrt{\frac{T}{\varrho }}.$$

It can be seen in the expression for *K*(*Ω*) that the presence of microstructure splits the dispersion relation for the string *ω* = *c*_1_*k* (*Ω* = *K*) into two branches, whose areas (in the first *K* - - *Ω* quadrant) are

**Figure 3.**
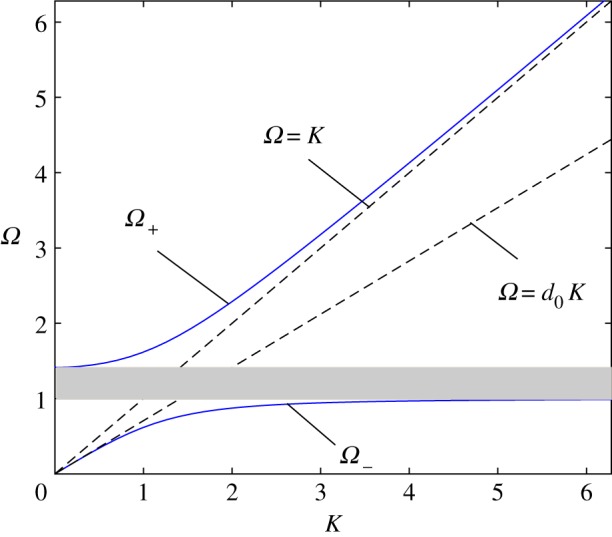
CN case. The two branches of the dispersion relation, *Ω* + (*k*) and *Ω* − (*k*). The asymptotes to the latter shown by dashed lines correspond to *k* → ∞ (*Ω* = *K*) and $k\to 0\;(\Omega =K/\sqrt{1+\varrho_{01}})$, respectively ($d_{0}=1/\sqrt{1+\varrho_{01}}$). The band gap is located in $\Omega_{-}(\infty )=1<\Omega <\sqrt{1+\varrho_{01}}=\Omega_{+}(0)$. The plot corresponds to ϱ_01_ = ϱ_0_/ϱ = 1. The shaded strip corresponds to the band gap. (Online version in colour.)

4.5 $$0\leq \Omega_{-}<1\;\mathrm{and}\;\sqrt{1+\varrho_{01}}\leq \Omega_{+}<\infty ,$$

where *K* is imaginary within the band gap between them (figure 3).

**Figure 4.**
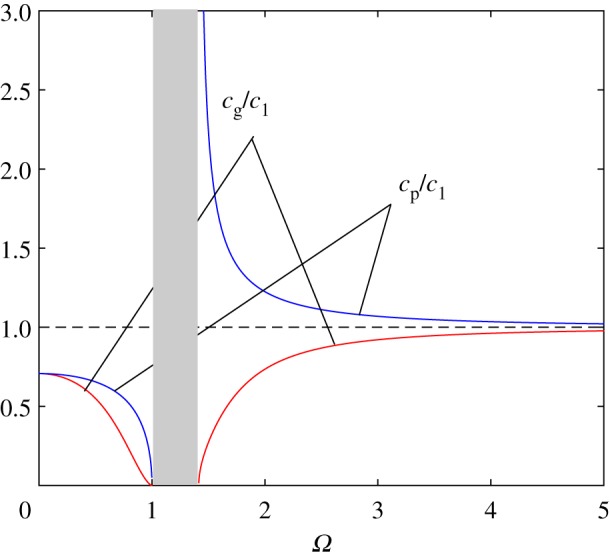
CN case. The group and phase velocities, *c*_*g*_ (red) and *c*_*p*_ (blue), outside the band gap *Ω* < 1 and $\sqrt{1+\varrho_{01}}<\Omega$. The plot corresponds to ϱ_01_ = ϱ_0_/ϱ = 1. (Online version in colour.)

The phase and group velocities are (see figure 4)

4.6 $$\left.\begin{array}{rl} c_{p} & =\frac{\omega }{k}=c_{1}\frac{\Omega }{K}=c_{1}\sqrt{\frac{1-\Omega^{2}}{1+\varrho_{01}-\Omega^{2}}}\\ \mathrm{and}\;\;c_{g} & =\frac{\;d\omega }{\;dk}=c_{1}\frac{\;d\Omega }{\;dK}=\frac{c_{1}^{2}}{c_{p}}\frac{{\left(1-\Omega^{2}\right)}^{2}}{{\left(1-\Omega^{2}\right)}^{2}+\varrho_{01}}. \end{array}\right\}$$

It is remarkable that, in both regions where the sinusoidal wave exists, the group velocity is below that in the free string, *c*_g_ < *c*_1_, and tends to zero as the frequency approaches a band gap boundary (figure 4).

### (b) Energy partitioning between the master body and the microstructure

For a sinusoidal wave

4.7 $$(u,v)=(A,A_{0})\;e^{i(\omega t-kx)}\;\mathrm{and}\;p_{1}=p_{2}=0,$$

it follows from (3.3) (with (3.2)) that

4.8 $$A=\left(1+\frac{G_{0}^{\mathrm{LF}}(i\omega ,k)}{\varkappa }\right)A_{0},\;\omega =\omega (k).$$

For the considered structured system with microstructure (the case CN), we obtain

4.9 $$A=(1-\Omega^{2})A_{0},\;\Omega =\frac{\omega }{\omega_{0}}.$$

The dispersion relation (3.5) for this particular structure takes the form (4.2) and the group velocity is computed in (4.6). The total energy density associated with the major structure and micro-substructures can be represented by

4.10 $$E_{\mathrm{total}}=E+E_{0}=\frac{1}{2}\varrho \omega^{2}A^{2}+\frac{1}{2}\varrho_{0}\omega^{2}A_{0}^{2}.$$

Note that the kinetic–potential energy partitioning is considered in Slepyan [43].

**Remark**

As follows from (4.9), in the limit (*ω* → *ω*_0_), the total energy settles in the microstructure ($E_{0}=E_{\mathrm{total}}$).

### (c) Continuously distributed locally interconnected oscillators (case CI)

With reference to (3.15) we have the dispersion relation

4.11 $$Tk^{2}-\varrho \omega^{2}+\frac{\varrho_{0}\omega_{0}^{2}(\varkappa_{0}k^{2}-\varrho_{0}\omega^{2})}{(\omega_{0}^{2}-\omega^{2})\varrho_{0}+\varkappa_{0}k^{2}}=0,$$

which in dimensionless form, as in (4.3), becomes

4.12 $$\begin{array}{r} \Omega^{4}-[1+\varrho_{01}+(1+c_{01}^{2})K^{2}]\Omega^{2}+(1+c_{01}^{2}\varrho_{01}+c_{01}^{2}K^{2})K^{2}=0,\;c_{01}=\frac{c_{0}}{c_{1}}\;\mathrm{and}\;c_{0}=\sqrt{\frac{\varkappa_{0}}{\varrho_{0}}}. \end{array}$$

In this case, there is no band gap in the sense that for any small *c*_01_ > 0 there exists a wavenumber k_*_(*c*_01_) such that for *k* > k_*_ there exist sinusoidal waves of any frequency. However, k_*_ → ∞ as *c*_01_ → 0. Thus, the dispersion dependence tends to that for *c*_01_ = 0 non-uniformly.

In this connection, we recall that each of the considered models, i.e. three-dimensional elasticity and the models of string and elastic rod, beam and plate, have their own inherent limit, upper for the wavenumber and lower for the wavelength. Thus, for a small *c*_01_ > 0, there is a range of frequencies over which (in contrast to the case *c*_01_ = 0) a more adequate theory is needed.

Note that for *c*_0_ = 0 this relation corresponds to the non-connected mass system, whereas for *c*_0_ = *c* this expression reduces to

4.13 $$\Omega =\Omega_{-}=\pm K\;\mathrm{and}\;\Omega =\Omega_{+}=\pm \sqrt{1+\varrho_{01}+K^{2}}.$$

Generally, the frequency corresponding to the dispersion relation (4.12) differs from that in (4.3) qualitatively and also quantitatively. In particular, if *c*_0_ > 0 the band gap no longer exists; however, for small *c*_01_, there exists a band of only short waves, as short as *c*_01_ is small.

**Figure 5.**
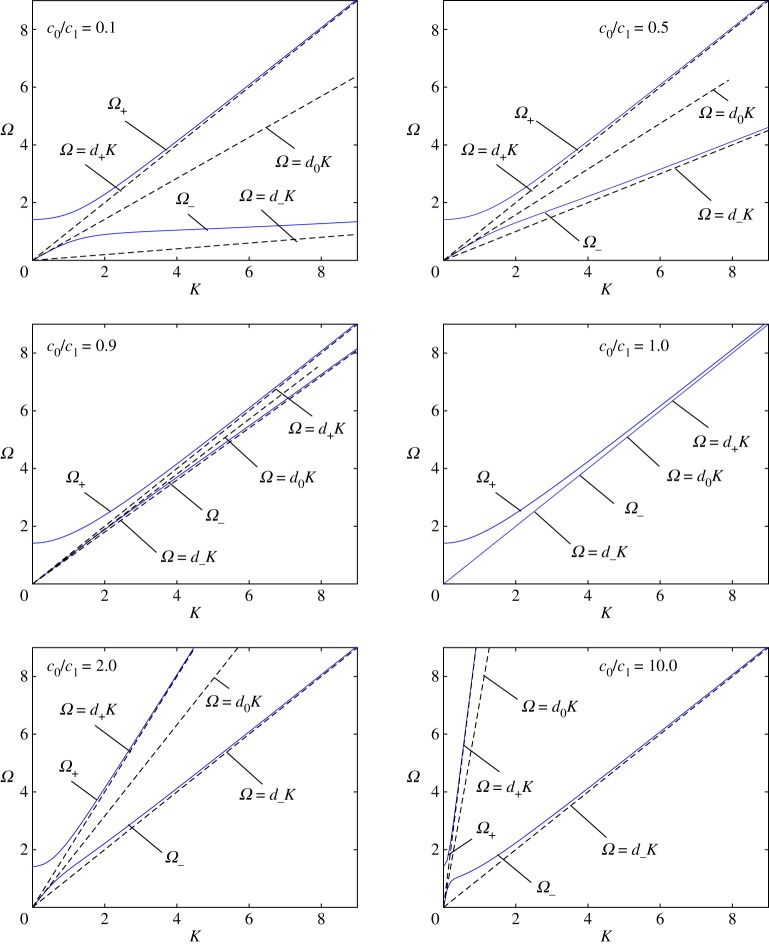
CI case. Continuously distributed interconnected oscillators. The dispersion curves (blue full curves) and the tangent lines (dashed lines) are plotted in accordance with (4.12) for six values of the attached-to-master structures speed ratio *c*_01_ = *c*_1_/*c*_0_. (Online version in colour.)

The dispersion dependences plotted in accordance with (4.12) are presented in figure 5 for ϱ_0_ = ϱ and some values of *c*_01_. The dashed lines correspond to the following asymptotes:

4.14 $$\Omega_{+}∼d_{+}K,\;\Omega_{-}∼d_{-}K,\;K\to \infty ,\;\text{and}\;\Omega_{-}∼d_{0}K,\;K\to 0,$$

where the constants involved are

$$d_{+}=\max\{1,c_{01}^{2}\},\;d_{-}=\frac{c_{01}^{2}}{d_{+}}\;\mathrm{and}\;d_{0}=\sqrt{\frac{1+c_{01}^{2}\varrho_{01}}{1+\varrho_{01}}}.$$

## 5. String with microstructure distributed discretely

We now consider a discrete set of oscillators, connected to the continuous string at *x* = *an*, *n* = 0,  ± 1, …, and the corresponding Floquet waves. Note that discreteness introduces additional frequencies, related to the oscillations of a string section between neighbouring contact points:

5.1 $$\omega_{n}=\frac{n\pi c_{1}}{a}.$$

Dispersion of waves is discussed in this section for two cases: discretely distributed oscillators not connected to each other (DN) and discretely distributed oscillators connected to each other (DI).

### (a) Discretely distributed oscillators not connected to each other (case DN)

Referring to (3.9), (3.16) and (4.1), we can write the following equation:

5.2 $$\left.\begin{array}{rl} & (Tk^{2}+\varrho s^{2})u^{\mathrm{LF}}=p_{1}^{\mathrm{LF}}+h^{\mathrm{LFd}}\\ \mathrm{with}\; & h^{\mathrm{LFd}}=-R^{\mathrm{LFd}}u^{\mathrm{LFd}}+\frac{\omega_{0}^{2}}{s^{2}+\omega_{0}^{2}}p_{2}^{\mathrm{LFd}},\;R^{\mathrm{LFd}}=\frac{m_{0}\omega_{0}^{2}s^{2}}{s^{2}+\omega_{0}^{2}}. \end{array}\right\}$$

It contains both the coupled continuous and discrete Fourier transforms, and we use the relationship (3.12) to obtain the discrete transform for the master body as for the microstructure. We thus have the equation as

5.3 $$u^{\mathrm{LFd}}=\left(-R^{\mathrm{LFd}}u^{\mathrm{LFd}}+\frac{\omega_{0}^{2}}{s^{2}+\omega_{0}^{2}}p_{2}^{\mathrm{LFd}}\right)\Sigma (s,k)+\Sigma_{p_{1}}(s,k),$$

where

5.4 $$\left.\begin{array}{rl} \Sigma (s,k) & =\frac{1}{a}\sum_{n=-\infty }^{\infty }{\left[\varrho s^{2}+T{\left(k+\frac{2\pi n}{a}\right)}^{2}\right]}^{-1}=\frac{\sinh(as/c_{1})}{2\varrho c_{1}s[\cosh(as/c_{1})-\cos(ak)]}\\ \mathrm{and}\;\Sigma_{p_{1}}(s,k) & =\frac{1}{a}\sum_{n=-\infty }^{\infty }{\left[\varrho s^{2}+T{\left(k+\frac{2\pi n}{a}\right)}^{2}\right]}^{-1}p_{1}^{\mathrm{LF}}\left(k+\frac{2\pi n}{a},s\right). \end{array}\right\}$$

Note that the above result can also be derived by dividing the domain into sections with subsequent conjugation. A straightforward technique based on (3.12) allows us to avoid such complications, from having to consider solutions for the continuous segments and match them on the discrete set of separating points. So in a one-dimensional case, the discussed method appears preferable. But its advantage is especially pronounced in two- and three-dimensional transforms, where the conventional procedure is of limited use [39].

Thus, we have an explicit expression for *u*^LFd^ as

5.5 $$u^{\mathrm{LFd}}=\frac{1}{1+R^{\mathrm{LFd}}(s)\Sigma (s,k)}\left(\Sigma_{p_{1}}(s,k)+\frac{\omega_{0}^{2}}{s^{2}+\omega_{0}^{2}}\Sigma (s,k)p_{2}^{\mathrm{LFd}}\right).$$

By letting *s* = i*ω* in the homogeneous equation

5.6 $$1+R^{\mathrm{LFd}}(i\omega )\Sigma (i\omega ,k)=0,$$

we obtain the dispersion relation using notation (4.4) for the normalized wavenumber and frequency *K* and *Ω* as used in the previous section, and where ϱ_01_ = *m*_0_/(*a*ϱ):

5.7 $$\cos(\Omega_{0}K)=\cos(\Omega_{0}\Omega )-P\sin(\Omega_{0}\Omega ),\;P=\frac{\varrho_{01}\Omega_{0}\Omega }{2(1-\Omega^{2})},\;\Omega_{0}=\frac{a\omega_{0}}{c_{1}},\;\Omega_{0}K=ak.$$

Note that the frequencies of the free oscillations of the string fixed at the reference points (*x* = *na*,  *n* = 0, 1, …) are *ω*_1*m*_ = *ω*_1_*m*, *ω*_1_ = *πc*_1_/*a*, *m* = 1, 2, …. It follows then that *Ω*_0_ is the multiplied-by-*π* ratio of the oscillator frequency to the main frequency of the string, *Ω*_0_ = *πω*_0_/*ω*_1_. Moreover, the dispersion relation (5.7) is 2*π*/*ω*_0_-periodic, *Ω*(*K*) = *Ω*(*K* + 2*π*/*Ω*_0_) and has an infinite set of branches.

**Figure 6.**
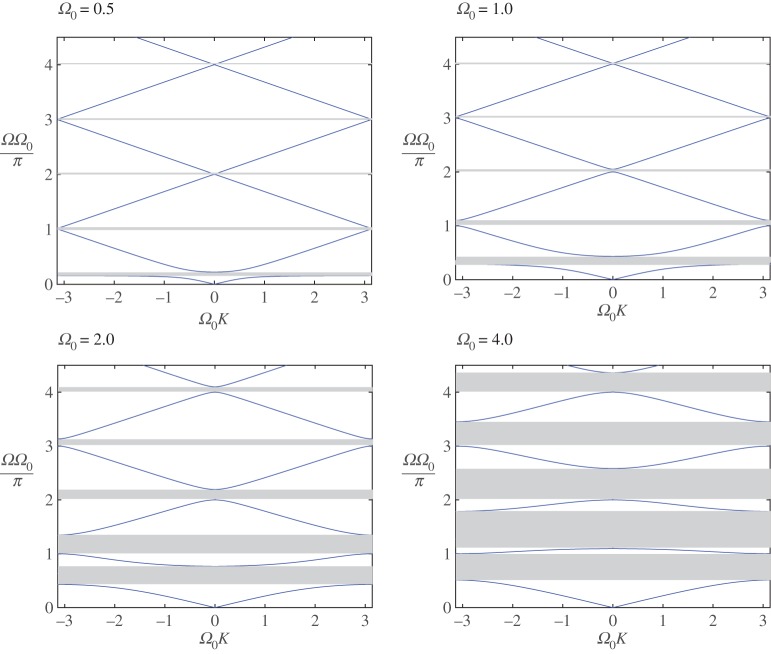
DN case. Discretely placed non-connected oscillators. The dispersion curves for four different values of *Ω*_0_ and ϱ_01_ = 1.0. (Online version in colour.)

In figure 6, we show how the dispersion relation evolves with change of *Ω*_0_, especially how the band gap width depends on this parameter. Asymptotically, as the dispersion diagram branch number, *m* = 1, 2, …, grows, the latter is situated in the strip (*Ω*^−^_*m*_, *Ω*^+^_*m*_), where

5.8 $$\Omega_{m}^{\pm }=\frac{\pi m}{\Omega_{0}}\left(1\pm \frac{\varrho_{01}\Omega_{0}^{2}}{\pi^{2}m^{2}-\Omega_{0}^{2}}\right).$$

### (b) Connected structured by-pass waveguide (case DI)

We analyse here the waves that occur in a structure consisting of an elastic continuous string, which is connected through vertical massless springs to a discrete set of masses; these masses are also connected by horizontal massless springs, as illustrated in figure 2.

For a variety of values of the physical parameters, we show that the dispersion of waves can be controlled in order to create ‘slow waves’ within intervals of given frequencies, i.e. in the desired frequency range, the group velocity can be reduced as required.

As in figure 2, the masses are interconnected, and the horizontal discrete chain is assumed to be under a tensile force *T*_0_. Referring to (3.9), (3.17) and (4.1), we have

5.9 $$\left.\begin{array}{rl} & (Tk^{2}+\varrho s^{2})u^{\mathrm{LF}}=p_{1}^{\mathrm{LF}}+h^{\mathrm{LFd}},\;h^{\mathrm{LFd}}=-R^{\mathrm{LFd}}u^{\mathrm{LFd}}+\frac{\varkappa p_{2}^{\mathrm{LFd}}}{G_{0}^{\mathrm{LFd}}+\varkappa }\\ \mathrm{and}\;\; & R^{\mathrm{LFd}}=\frac{\varkappa G_{0}^{\mathrm{LFd}}}{\varkappa +G_{0}^{\mathrm{LFd}}},\;G_{0}^{\mathrm{LFd}}=m_{0}s^{2}+2T_{0}(1-\cos ak). \end{array}\right\}$$

We continue to use the notation $R^{\mathrm{LFd}}$ and note that, in contrast with the previous notation, where $R^{\mathrm{LFd}}=R^{\mathrm{LFd}}(s)$, in the case under consideration $R^{\mathrm{LFd}}=R^{\mathrm{LFd}}(s,k)$. Thus

5.10 $$u^{\mathrm{LF}}=\frac{p_{1}^{\mathrm{LF}}}{Tk^{2}+\varrho s^{2}}-\frac{h^{\mathrm{LFd}}}{Tk^{2}+\varrho s^{2}}.$$

The ‘continuous-to-discrete converter’ (3.11) yields

5.11 $$(1+R^{\mathrm{LFd}}\Sigma )u^{\mathrm{LFd}}=\frac{\varkappa p_{2}^{\mathrm{LFd}}}{G_{0}^{\mathrm{LFd}}+\varkappa }\Sigma +\Sigma_{p_{1}},$$

where *Σ* = *Σ*(*s*, *k*) and *Σ*_*p*_1__ = *Σ*_*p*_1__(*s*, *k*) are defined in (5.4).

The dispersion relation for the Floquet–Bloch waves is derived similarly to (5.6) and has the form

5.12 $$1+R^{\mathrm{LFd}}(i\omega ,k)\Sigma (i\omega ,k)=0.$$

**An alternative derivation of the dispersion relation.** For the convenience of the reader, we outline the derivation of the dispersion relation, without using the notion of a quasi-periodic Green's function.

For the waves in the elastic string, loaded by continuously distributed as well as periodically distributed forces, the governing equation has the form

5.13 $$\varrho \frac{\partial^{2}u(x,t)}{\partial t^{2}}-E\frac{\partial^{2}u(x,t)}{\partial x^{2}}=\sum_{n=-\infty }^{\infty }Q_{n}(t)\delta (x-an)+p_{1}(x,t),\;Q_{n}=-\varkappa [u(an,t)-v_{n}(t)].$$

The attached-mass equation of motion is given by

5.14 $$\frac{\;d^{2}v_{n}}{\;dt^{2}}=-\frac{Q_{n}}{m_{0}}-\omega_{d}^{2}(v_{n-1}-2v_{n}+v_{n+1})+\frac{P_{n}(t)}{m_{0}},\;\omega_{d}=\sqrt{\frac{d}{m_{0}}}.$$

Here *c* is the stiffness of the springs linking the masses in the chain, while ϰ is the stiffness of each spring bonding the chain mass to the string.

Laplace transform over *t* yields

5.15 $$s^{2}v_{n}^{L}=\omega_{0}^{2}(u^{L}(an,s)-v_{n}^{L})-\omega_{d}^{2}(v_{n-1}^{L}-2v_{n}^{L}+v_{n+1}^{L})+\frac{P_{n}^{L}}{m_{0}},\;\omega_{0}=\sqrt{\frac{\varkappa }{m_{0}}}.$$

By application of the discrete Fourier transform, we have, after some algebra,

5.16 $$v^{\mathrm{LFd}}=\frac{\omega_{0}^{2}u^{\mathrm{LFd}}}{s^{2}+\omega_{0}^{2}-2\omega_{d}^{2}(1-\cos(ak))}+\frac{P^{\mathrm{LFd}}}{m_{0}(s^{2}+\omega_{0}^{2}-2\omega_{d}^{2}(1-\cos(ak)))}$$

and

5.17 $$Q^{\mathrm{LFd}}(s)=\frac{\varkappa (s^{2}-2\omega_{d}^{2}(1-\cos(ak)))u^{\mathrm{LFd}}}{s^{2}+\omega_{0}^{2}-2\omega_{c}^{2}(1-\cos(ak))}-\frac{\omega_{0}^{2}P^{\mathrm{LFd}}}{s^{2}+\omega_{0}^{2}-2\omega_{d}^{2}(1-\cos(ak))}.$$

Here, as above,

$$u^{\mathrm{LFd}}(ak,s)={\left(u^{L}(an,s)\right)}^{\mathrm{Fd}}.$$

Applying Laplace and Fourier transforms to equation (5.13), we obtain

5.18 $$u^{\mathrm{LF}}(k,s)=\frac{1}{\varrho s^{2}+Ek^{2}}(-\varkappa Bu^{\mathrm{LFd}}(ak,s)+CP^{\mathrm{LFd}}(ak,s)+p_{1}^{\mathrm{LF}}(k,s)),$$

where

5.19 $$B(s,k)=\frac{s^{2}-2\omega_{d}^{2}(1-\cos(ak))}{s^{2}+\omega_{0}^{2}-2\omega_{d}^{2}(1-\cos(ak))}\;\mathrm{and}\;C(s,k)=\frac{\omega_{0}^{2}}{s^{2}+\omega_{0}^{2}-2\omega_{d}^{2}(1-\cos(ak))}.$$

Finally, following similar reasoning to that employed in (5.3) and (5.4), we obtain

5.20 $$\begin{array}{rl} u^{\mathrm{LFd}} & =(-\varkappa Bu^{\mathrm{LFd}}(ak,s)+CP^{\mathrm{LFd}}(ak,s))\frac{1}{a}\sum_{n=-\infty }^{\infty }\frac{1}{\varrho s^{2}+E{\left(k+2\pi n/a\right)}^{2}}\\ & \;+\frac{1}{a}\sum_{n=-\infty }^{\infty }\frac{p_{1}^{\mathrm{LF}}(k+2\pi n/a,s)}{\varrho s^{2}+E{\left(k+2\pi n/a\right)}^{2}}. \end{array}$$

For the homogeneous problem, we have

5.21 $$s\sum_{n=-\infty }^{\infty }{\left[\varrho s^{2}+E{\left(k+\frac{2\pi n}{a}\right)}^{2}\right]}^{-1}=-a\frac{s}{\varkappa B(s,k)},$$

and by summation of the left-hand side we obtain

5.22 $$\frac{c\sinh(sa/c)}{2E(\cosh(sa/c)-\cos(ak))}=-\frac{s}{\varkappa B(s,k)}.$$

The dispersion relation follows from (5.22) by substituting i*ω* for *s*, using the same independent variables as in (5.7):

5.23 $$\frac{\varrho_{01}}{2}\frac{\Omega_{0}\sin(\Omega_{0}\Omega )}{\Omega (\cos(\Omega_{0}\Omega )-\cos(\Omega_{0}K))}=\frac{1-\Omega^{2}-2\Omega_{d}^{2}(1-\cos(\Omega_{0}K))}{\Omega^{2}+2\Omega_{d}^{2}(1-\cos(\Omega_{0}K))},\;\Omega_{d}=\frac{\omega_{d}}{\omega_{0}}.$$

## 6. ‘Slow’ waves for frequency regimes close to a resonance of the DN system

As mentioned above, it is interesting to analyse how the system behaves in the vicinity of *Ω*_0_ = *π* and *Ω* = 1. We note that *Ω*_0_ = *π* corresponds to the particular case when the first frequencies of the microstructure and the string section of the major structure (between neighbouring oscillators) coincide. We may check by inspection that there is no real wavenumber corresponding to the frequency *Ω* = 1 (see dispersion equation (5.7)). Nevertheless, we will show that a real solution exists for any value of *Ω*_0_≠*π*.

**Figure 7.**
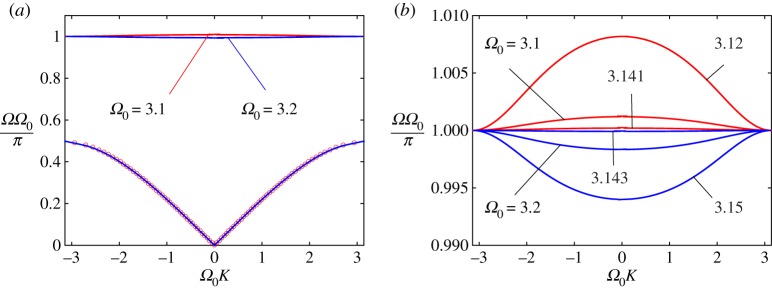
DN model for ϱ_01_ = 1.0. (*a*) The lower two branches are for two values of the parameter *Ω*_0_ close to *π* (*Ω*_0_ = 3.1 < *π*, *Ω*_0_ = 3.2 > *π*). (*b*) Only the second branches are shown for some values of *Ω*_0_ close to *π* from both sides. (Online version in colour.)

In figure 7*a*, we present the first two branches of the dispersion diagram for two values of the parameter *Ω*_0_ close to the limiting case (*Ω*_0_ = *π*) from above and below. Here the relative mass densities ϱ_01_ = 1.

We observe that, as predicted, the first branches of the dispersion diagram exist for any values in the neighbourhood of *Ω*_0_ = *π* and they are visually indistinguishable. The value of *Ω*_0_ = *π* separates the signs of the group velocity in the second branch (compare the last two graphs in figure 6). As can be observed for frequencies close to this value, practically no waves propagate along the string. At the same time, oscillators connected to it vibrate almost independently of each other (compare figure 7*b*).

In figure 7b, we demonstrate that the optical mode converges to the limiting case *Ω*(*K*) = 1 when *Ω*_0_ → *π*, while the mode does not exist (there is no real wavenumber when *Ω*_0_ = *π*). However, it does exist for any value of *Ω*_0_≠*π*.

**Remark**

To explain this phenomenon in more detail, we note that the dispersion relation in (5.7) defines a real function *K* = *K*(*Ω*, *Ω*_0_) in the neighbourhood of a point (*Ω*, *Ω*_0_) in the domain *Ω* > 0, *Ω*_0_ > 0. The points (*Ω*, *Ω*_0_) = (1, *mπ*) (*m* = 1, 2, …) are essentially singular points for this function, in the sense that the limits do not exist, but the different real limits do exist along different rays within a sector in the (*Ω*, *Ω*_0_) plane while different complex limits exist if any ray is considered.

**Figure 8.**
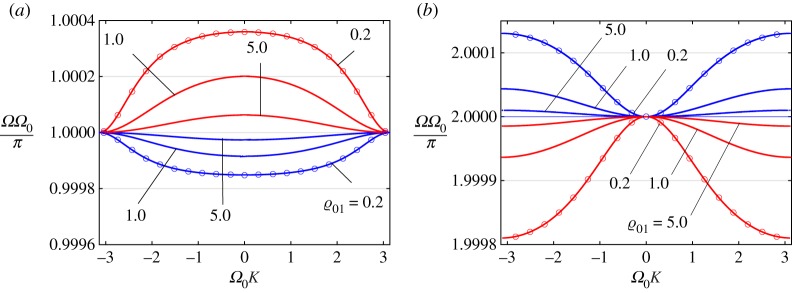
DN model. Dispersion dependences for different values of the relative density (ϱ_01_ = 0.2, 1.0, 5.0). (*a*) The second branch (optical mode) for two values of the parameter *Ω*_0_ close to *π* (*Ω*_0_ = 3.1, 3.2). (*b*) The third branch for two values of the parameter *Ω*_0_: the red colour corresponds to *Ω*_0_ = 6.282 (the curves lie above unity) and the blue colour corresponds to *Ω*_0_ = 6.286 (the curves lie below unity). Circles correspond to values of *Ω* computed using asymptotic relationship (6.1). (Online version in colour.)

In figure 8, we show that this affects how the second (third) branch behaves in response to changes in the ratio of the masses of the structures near the points (1, *π*) and (1, 2*π*). Specifically, for different values of the relative density (ϱ_01_ = 0.2, 1.0, 5.0) we present, in figure 8*a*, the second branch (optical mode) for two values of the parameter *Ω*_0_ close to *π* (*Ω*_0_ = 3.1, 3.2) and, in figure 8*b*, the third branch for two values of the same parameter, *Ω*_0_ = 6.282 (the curves lie above unity) and *Ω*_0_ = 6.286 (the curves lie below unity). Circles correspond to values of *Ω* as computed using asymptotic relationship (6.1).

Note that the optical branch can be approximated for the value of *Ω*_0_ ≈ *π* in the following form:

6.1 $$\Omega =\frac{8\cos^{2}(\Omega_{0}K/2)+\pi^{2}\varrho_{01}}{8\cos^{2}(\Omega_{0}K/2)+\pi \Omega_{0}\varrho_{01}}.$$

Moreover, note that

6.2 $$\Omega {|}_{K=0}=\frac{8+\pi^{2}\varrho_{01}}{8+\pi \Omega_{0}\varrho_{01}},\;\Omega {|}_{\Omega_{0}K=\pm \pi }=\frac{\pi }{\Omega_{0}}\;\mathrm{and}\;[\Omega ]=\frac{8(\Omega_{0}-\pi )}{\Omega_{0}(8+\pi \Omega_{0}\varrho_{01})}.$$

In the same manner, we can analyse the third branch of the dispersion diagram in the case *Ω*_0_ ≈ 2*π*. Here the approximate formula is found to be

6.3 $$\Omega =\frac{4\sin^{2}(\Omega_{0}K/2)+2\pi^{2}\varrho_{01}}{4\sin^{2}(\Omega_{0}K/2)+\Omega_{0}\pi \varrho_{01}},$$

and

6.4 $$\Omega {|}_{K=0}=\frac{2\pi }{\Omega_{0}},\;\Omega {|}_{\Omega_{0}K=\pm \pi }=\frac{4+2\pi^{2}\varrho_{01}}{4+\Omega_{0}\pi \varrho_{01}}\;\mathrm{and}\;[\Omega ]=\frac{4(2\pi -\Omega_{0})}{\Omega_{0}(4+\Omega_{0}\pi \varrho_{01})}.$$

The corresponding results are presented in figure 8*b*.

We can also deliver similar formulae for the higher resonance cases (*m* = 3, 4, …):

6.5 $$\Omega =\frac{\pi m}{\Omega_{0}}+\frac{4(\Omega_{0}-\pi m)\left[1-{\left(-1\right)}^{m}\cos(\Omega_{0}K)\right]}{\Omega_{0}(\Omega_{0}m\varrho_{01}+4)\left[1-{\left(-1\right)}^{m}\cos(\Omega_{0}K)\right]}$$

and

6.6 $$\Omega_{m}^{\left(1\right)}=\frac{\pi m}{\Omega_{0}},\;\Omega_{m}^{\left(2\right)}=\frac{\pi m^{2}\varrho_{01}+8}{\Omega_{0}m\pi \varrho_{01}+8}\;\mathrm{and}\;[\Omega ]=\frac{{\left(-1\right)}^{m}8(\pi m-\Omega_{0})}{\Omega_{0}(\Omega_{0}m\varrho_{01}+8)}.$$

To conclude, when *Ω*_0_ is close to *π*, while *Ω* is close to unity, the energy settles in the microstructure, while slowly transferring along the master body. There are no solutions in cases where *Ω* = 1 and *Ω*_0_ = *πm* (*m* = 1, 2, 3, …), when the oscillators are in the resonance mode. Note that all of those singular points (*Ω*, *Ω*_0_) = (1, *πm*) are situated in the band gaps (figure 6).

**Figure 9.**
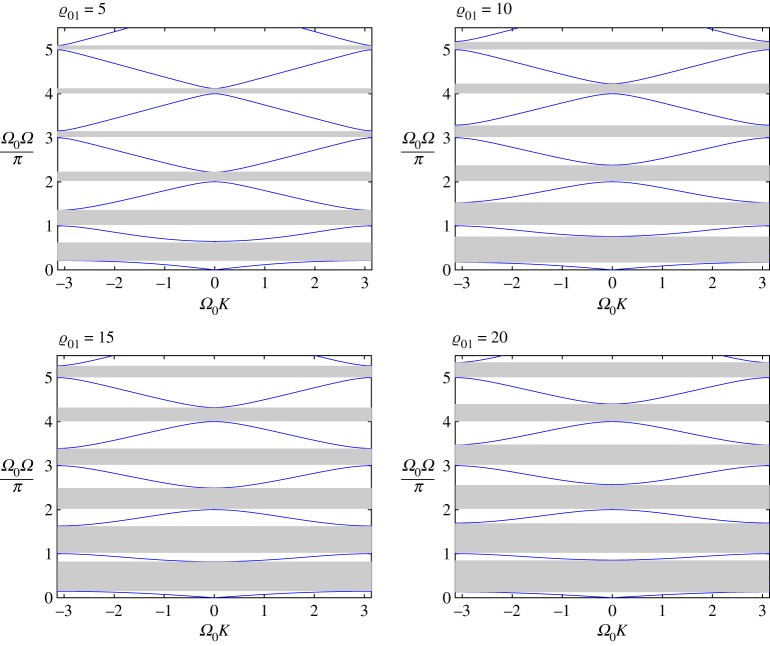
DN model. The dispersion curves for different values of the parameter ϱ_01_ = 5.0, 10, 15, 20 for *Ω*_0_ = 1.0. Note that the case ϱ_01_ = 1.0 and *Ω*_0_ = 1.0 has also been presented in figure 6. (Online version in colour.)

In figure 9, we present dispersion diagrams for different values of the mass ratio ϱ_01_ = 5, 10, 15, 20 of the structures for the same value of *Ω*_0_ = 1.0. To enhance the scope of the analysis, we can also compare those results with figure 6, where ϱ_01_ = 1. It is clear that the sensitivity to this latter parameter is much weaker than that to *Ω*_0_.

## 7. Comparison of the continuous (CN) and discrete (DN) models

Finally, it is important to compare the CN and DN models. We might expect that the latter model would under some conditions be ‘close’ in some sense to the former (considered as its homogenized version). As a result, the first two branches of the dispersion relation (4.3) in the case CN should be similar to those in (5.7).

**Figure 10.**
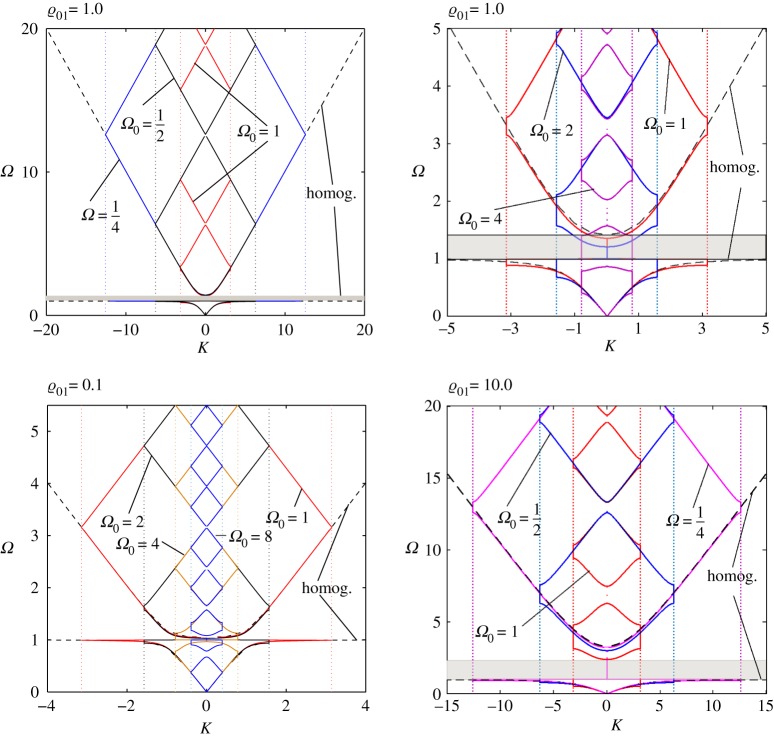
CN model versus DN model. The dispersion curves for different values of the ratio ϱ_01_ = 0.1, 1.0, 10.0. (Online version in colour.)

In figure 10, we present some results in the original variables for comparison of the discussed cases. The homogenized model (represented by the CN model) is shown by the dashed line, while by solid lines of different colours we have depicted the body with the embedded and disconnected structure (the DN model). We can see that in some cases the first two branches of the latter can be confidently approximated by the corresponding *homogenized* continuous structure represented by the CN model. However, the accuracy of this approximation depends on the ratios of the densities/masses and the frequencies of the master and the embedded structures.

This coincidence (closeness) between the first two branches of the dispersion diagrams for the CN and DN models happens when

**Figure 11.**
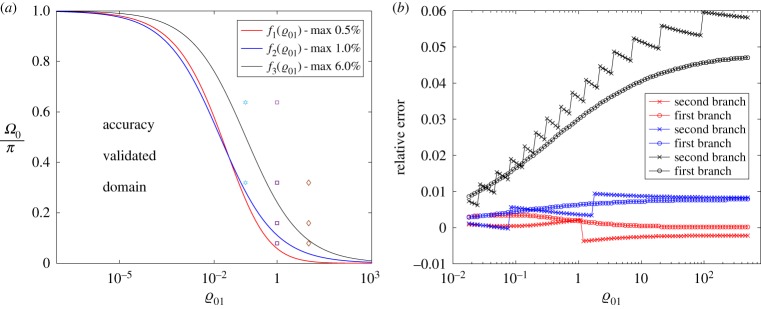
Deviation between the CN and DN models. (*a*) By different markers, we have depicted the different cases presented in figure 10. (*b*) The maximal relative deviation between the respective branches along the curves shown in (*a*) (the colours correspond). (Online version in colour.)

7.1 $$\Omega_{0}<f(\varrho_{01}),$$

where *f* is a function defined numerically to guarantee that the difference between the homogenized problem and the accurate problem is within a prescribed tolerance. We have evaluated the following three functions, *f*_*j*_ (*j* = 1, 2, 3) that guarantee the closeness between the first two branches with an accuracy of 0.5%, 1% and 6%, respectively (figure 11):

7.2 $$f_{1}(x)=\frac{\pi }{{\left(1+\pi \sqrt{x}\right)}^{2}},\;f_{2}(x)=\frac{\pi }{1+8\sqrt{x}}\;\mathrm{and}\;f_{3}(x)=\frac{\pi }{1+\pi \sqrt{x}}.$$

There is an interesting exceptional case here, when the master structure is practically massless (ϱ → 0) (figure 11*a*). Then *Ω*_0_ → 0, while ϱ_01_ → ∞. Moreover,

$$\Omega_{0}^{2}\varrho_{01}=b\equiv a\omega_{0}^{2}\frac{m_{0}}{T}.$$

The dispersion diagram for this specific limiting case is then

$$\Omega =\sqrt{\frac{2(1-\cos(ak))}{2+b-2\cos(ak)}},$$

and only one branch of the dispersion diagram exists in the case CN, bounded from above by the value $1/\sqrt{1+b/4}$.

Note that the higher branches in the dispersion diagrams can also be approximated by asymptotic techniques [44–47].

## 8. High-contrast systems DI: wave dispersion and resonances

The dispersion equation (5.12) has been solved numerically. Note that equation (5.23) also allows us to explicitly determine the wavenumber expressed as a function of the frequency (compare (5.7)). However, the right-hand side of the respective formula is rather cumbersome and we do not present it here. In the case *Ω*_*d*_ = 0, equation (5.23) coincides with (5.7) as we would expect. Interestingly, taking the interaction of the main continuous waveguide with the discrete structure into account, we observe that the asymptotic formulae (5.8) are still valid with an accuracy of *O*(*m*^−2^) as *m* → ∞.

The presence of the discrete system of connected resonators leads to the formation of stop bands in the dispersion diagram, as well as the formation of slow waves. The dispersion properties strongly depend on the relative inertia of embedded resonators, characterized by the parameter ϱ_01_ as well as the relative spectral properties of the embedded discrete structure versus the continuous waveguide, characterized by the parameters *Ω*_0_ and *Ω*_*d*_.

**Figure 12.**
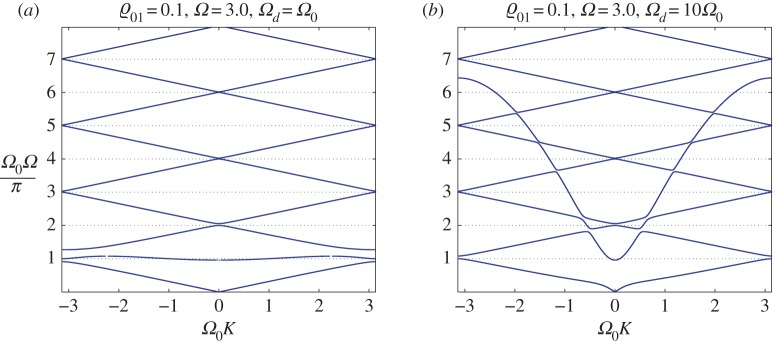
DN model. Dispersion curves for the high-contrast case of a system of connected resonators attached to an infinite inertial spring: (*a*) the influence of the resonators is apparent in the low-frequency regime, where slow waves are observed; and (*b*) the region of influence for elastic resonators has been widened with the increase of *Ω*_*d*_/*Ω*_0_. (Online version in colour.)

Figure 12*a*,*b* corresponds to ϱ_01_ = 0.1, and when *Ω*_0_ = *Ω*_*d*_ = 3.0, we observe in figure 12*a* that, at higher frequencies, the dispersion diagram is very close to that of a homogeneous string, without crossing of the dispersion curves. At an interval of frequencies adjacent to the origin, the change of dispersion properties is definitive, as provided by the interaction of the continuous waveguide with the embedded discrete structure: an interval of slow waves has been formed adjacent to a full stop band. We also note that there are no horizontal dispersion lines, as discussed earlier in the text. Given an unchanged ϱ_01_ and *Ω*_0_ and a significantly increased *Ω*_*d*_ = 10*Ω*_0_ (*Ω*_0_ = 3.0), we observe stronger coupling between the continuous waveguide and the discrete structure, which is reflected in the change of dispersion pattern shown in figure 12*b*, where a wider interval of frequencies has been affected.

**Figure 13.**
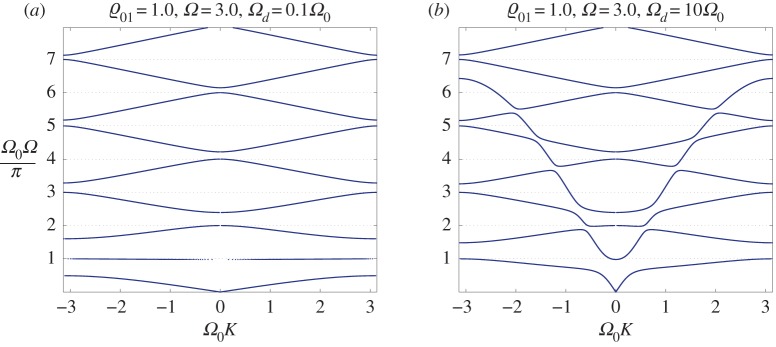
DN model. Dispersion curves for the case of an increased inertia of connected resonators attached to an infinite elastic string: (*a*) stop bands have been formed at higher frequencies, slow waves are observed in a low-frequency range; and (*b*) a significant increase in the influence of the embedded resonators as *Ω*_*d*_/*Ω*_0_ increases. (Online version in colour.)

An increase in the inertia of the embedded discrete structure leads to a significant change in the dispersion properties and the opening of band gaps in a higher frequency range, as shown in figure 13*a*,*b*. For these diagrams, we have chosen ϱ_01_ = 1.0, while *Ω*_0_ = 3.0. In figure 13*a*, we use a relatively small *Ω*_*d*_ = 0.1*Ω*_0_, and figure 13*b* corresponds to a significantly greater value of *Ω*_*d*_ = 10*Ω*_0_. As in figure 12*a*, the lower frequency range, illustrated in figure 13*a*, includes a dispersion curve corresponding to slow waves, while figure 13*b* appears to be similar to figure 12*b*, as expected for large values of the parameter *Ω*_*d*_, where the proposed change in the inertial parameter ϱ_01_ does not appear dominant.

**Figure 14.**
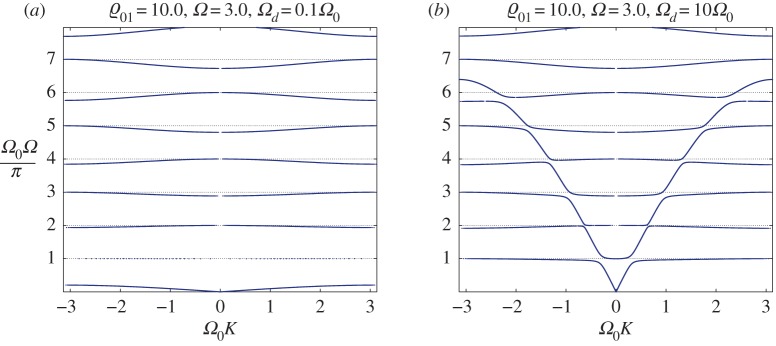
DN model. The case of high inertia of embedded resonators attached to an infinite elastic string: (*a*) slow waves are observed along the stop bands across a wide range of frequencies; and (*b*) low-frequency stop bands close down or reduce their width with the increase of the value *Ω*_*d*_/*Ω*_0_. (Online version in colour.)

By continuing to increase the inertial parameter ϱ_01_, we make a further significant change in the dispersion diagrams, as shown in figure 14*a*,*b*. An increased width of full stop bands in a higher frequency range is the characteristic feature observed for the case of the discrete embedded structure being supplied with significantly enhanced inertia properties. This is especially pronounced in figure 14*a*, where the values of the parameters are ϱ_01_ = 10, *Ω*_0_ = 3.0, *Ω*_*d*_ = 0.1*Ω*_0_. On this dispersion diagram, we observe that ‘slow waves’ appear in a wide frequency interval adjacent to the origin. The dispersion properties change dramatically as the parameter *Ω*_*d*_, characterizing the spectral properties of the structure, is increased so that *Ω*_*d*_ = 10*Ω*_0_, as shown in figure 14*b*. The combined waveguide now supports slow waves as well as waves of higher group velocity, and in this case the discrete connected structure is acting as a ‘wave by-pass’ channel, which diverts the energy of vibration from the continuous string to the discrete structure.

## 9. Bernoulli–Euler beam as the master body

In this section, the master body is described by a fourth-order differential equation, and the Laplace–Fourier transform of the inverse Green's function for the elastic flexural beam is

9.1 $$G^{\mathrm{LF}}=Dk^{4}+\varrho s^{2},$$

where *D* is the bending stiffness of the beam and ϱ is the mass per unit length. Here we consider two cases: continuously distributed oscillators (CN) and discretely distributed oscillators with no interconnection (DN). The remaining cases (CI and DI) can be analysed analogously.

### (a) The case of continuously distributed oscillators (CN)

With reference to (3.4) and (3.14), we write the equation

9.2 $$\left(Dk^{4}+\left(1+\frac{\varrho_{01}\omega_{0}^{2}}{s^{2}+\omega_{0}^{2}}\right)\varrho s^{2}\right)u^{\mathrm{LF}}=p_{1}^{\mathrm{LF}}+\frac{\omega_{0}^{2}}{s^{2}+\omega_{0}^{2}}p_{2}^{\mathrm{LF}},$$

where *D* and ϱ are as above and ϱ_01_ = ϱ_0_/ϱ. The corresponding dispersion relation is

**Figure 15.**
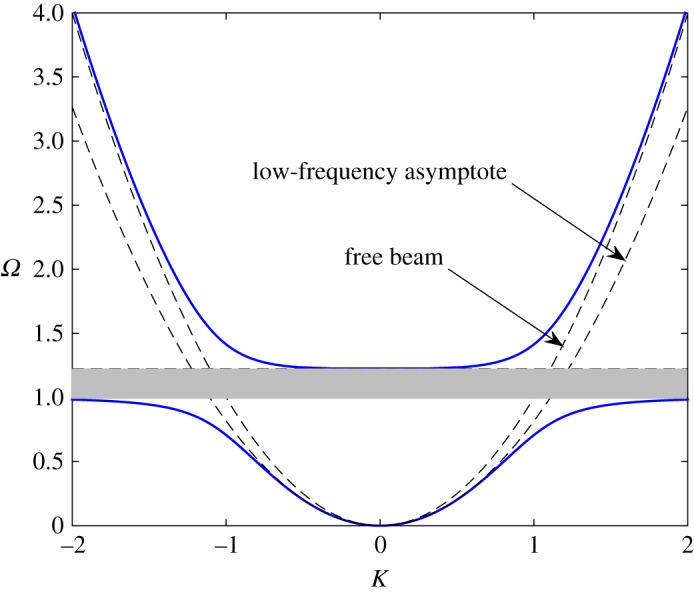
CN model. Dispersion diagram for a beam with continuously distributed oscillators, i.e. *Ω* as a function of *K* for ϱ_01_ = 0.5 (blue solid lines). There exists a band gap, $1<\Omega <\sqrt{1+\varrho_{01}}$, where the harmonic waves cannot propagate along the structure. The dependence for the free beam $K=\sqrt{\Omega }$ and the low-frequency asymptotics $K∼{\left(1+\varrho_{01}\right)}^{1/4}\sqrt{\Omega }$ as *Ω*≪1 are also shown (dashed lines). (Online version in colour.)

9.3 $$K_{b}={\left(1+\frac{\varrho_{01}}{1-\Omega^{2}}\right)}^{1/4}\Omega^{1/2},\;K_{b}={\left(\frac{D}{\varrho \omega_{0}^{2}}\right)}^{1/4}k,$$

and the function *Ω* versus *K* is plotted in figure 15.

Note that *K*_*b*_ = *K* is the same normalization as used above ($K=k/\sqrt{\Omega_{0}}$), with *Ω*_0_ = *ω*_0_/*c*_1_, $c_{1}=\sqrt{D/\varrho }$.

It can be seen that the group velocity in the equipped beam is below that in the free one, d*ω*/ d*k* < 2*k*. This shows that a structure such as a line of resonators attached to an elastic beam is a waveguide. Note that the band gap appears to be similar to that in the case of the string. This is due to the fact that it is defined by the frequency-dependent inertia (the mass and the added mass), which is the same for both cases.

Qualitatively, this effect is similar to the formation of the stop band that has been observed in the CN case of an extensible string being used as the master body (see figure 3). The obvious differences are of course observed due to the different orders of the dispersion equations in these two cases; the latter includes different asymptotics of *Ω*(*K*) when *K* → 0 and when *K* → ∞.

### (b) Discretely positioned oscillators on a flexural elastic beam (case DN)

In the case of discretely distributed oscillators, the equation becomes

9.4 $$\left.\begin{array}{rl} & (Dk^{4}+\varrho s^{2})u^{\mathrm{LF}}=p_{1}^{\mathrm{LF}}(k,s)+h^{\mathrm{LFd}}\\ \mathrm{with}\;\; & h^{\mathrm{LFd}}=-R^{\mathrm{LFd}}u^{\mathrm{LFd}}+\frac{\omega_{0}^{2}}{s^{2}+\omega_{0}^{2}}p_{2}^{\mathrm{LFd}},\;R^{\mathrm{LFd}}=\frac{m_{0}\omega_{0}^{2}s^{2}}{s^{2}+\omega_{0}^{2}}, \end{array}\right\}$$

and we have to deduce the continuous transform of *u*,

9.5 $$u^{\mathrm{LF}}=\frac{p_{1}^{\mathrm{LF}}(k,s)+h^{\mathrm{LFd}}}{Dk^{4}+\varrho s^{2}},$$

to the discrete version.

With reference to (3.12) (also see (5.3)) we find

9.6 $$u^{\mathrm{LFd}}=h^{\mathrm{LFd}}\Sigma_{\mathrm{beam}}+\frac{1}{a}\sum_{-\infty }^{\infty }\frac{p_{1}^{\mathrm{LF}}(k+2\pi n/a,s)}{D{\left(k+2\pi n/a\right)}^{4}+\varrho s^{2}}$$

where

9.7 $$\left.\begin{array}{rl} \Sigma_{\mathrm{beam}} & =\frac{1}{a}\sum_{n=-\infty }^{\infty }\frac{1}{D{\left(k+2\pi n/a\right)}^{4}+\varrho s^{2}}=\frac{a^{3}}{D}\Psi (ak,\psi )\\ \mathrm{and}\;\Psi (ak,\psi ) & =\sum_{n=-\infty }^{\infty }\frac{1}{{\left(ak+2\pi n\right)}^{4}-\delta^{4}\psi^{2}}\\ & =\frac{\sin(\delta \psi )[\cosh(\delta \psi )-\cos(ak)]-\sinh(\delta \psi )[\cos(\delta \psi )-\cos(ak)]}{4{\left(\delta \psi \right)}^{3}[\cos(\delta \psi )-\cos(ak)][\cosh(\delta \psi )-\cos(ak)]}, \end{array}\right\}$$

and *δ* = (ϱ*a*^4^/*D*)^1/4^ while $\psi =\sqrt{-is}$ and $\psi =\sqrt{\omega }\;(s=i\omega )$ in the case of the complex wave.

Thus, the dispersion relation is

9.8 $$\frac{\delta^{4}\omega_{0}^{2}\varrho_{01}\Omega^{2}}{1-\Omega^{2}}\Psi (ak,\sqrt{\omega })=1.$$

This can also be written in an equivalent form as

9.9 $$\frac{\varrho_{01}\delta^{4}\Omega^{2}}{1-\Omega^{2}}F(\delta \sqrt{\Omega },\;\delta K)=1,\;-\frac{\pi }{\delta }\leq K\leq \frac{\pi }{\delta },\;\Omega >0,$$

where we have taken into account the unified notation $\delta =a\sqrt{\Omega_{0}}$, *Ω*_0_ = *ω*_0_/*c*_1_, *Ω* = *ω*/*ω*_0_, ϱ_01_ = *m*_0_/(ϱ*a*) and $K=k/\sqrt{\Omega_{0}}$. Note that the dispersion relation is *π*/*δ*-periodic.

In the case where *δ* → 0 and ϱ_01_ is assumed fixed (the mass distribution remains the same), equation (9.9) when restricted to its leading asymptotic terms is reduced to

$$K^{4}=\Omega^{2}\frac{1+\varrho_{01}-\Omega^{2}}{1-\Omega^{2}}+O(\delta^{2}),\;\delta \to 0,$$

which naturally corresponds to (9.2) as the homogenization limit.

### (c) Continuous (CN) versus discrete (DN) model

**Figure 16.**
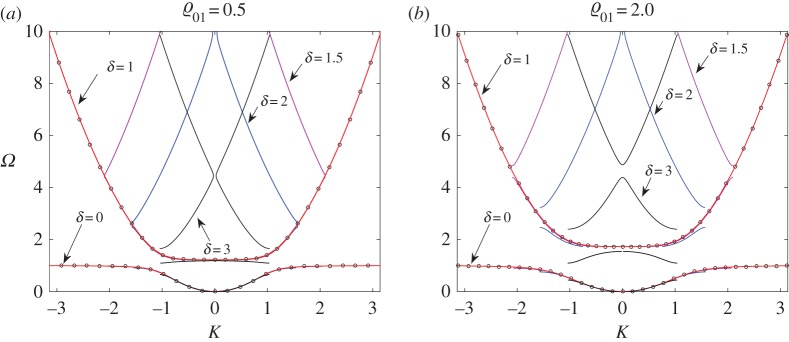
DN model. Dispersion diagrams for (*a*) ϱ_01_ = 0.5 and (*b*) ϱ_01_ = 2.0 for different values of *δ* = 1.0, 1.5, 2.0, 3.0 (depicted by the solid lines of different colours). By red markers, we show the limiting homogenization problem (*δ* = 0) corresponding to the beam with continuous distributed oscillators considered in the previous section (the CN model). (Online version in colour.)

Here we show how the results associated with the discrete formulation compare with those from the continuous approximation. In figure 16, we present the dispersion diagrams for the discrete model for different values of *δ* = 1.0, 1.5, 2.0, 3.0 (as depicted by the solid lines).

For small values of *δ*, we can approximate the discrete problem with the corresponding continuous analogy under an appropriate choice of problem parameters. However, an important question arises as to whether such a replacement can be quantitatively estimated to justify itself. We have already demonstrated how the techniques developed can help with this (compare figure 11). To make such a prediction, we now introduce two measures:

9.10 $$E_{C}^{\left(j\right)}=\max_{K\in [0,\pi /\delta ]}\{|\Omega_{\delta }^{\left(j\right)}(K)-\Omega_{0}^{\left(j\right)}(K)|{\left(\Omega_{0}^{\left(j\right)}(K)\right)}^{-1}\},\;j=1,2,$$

and

9.11 $$E_{L_{2}}^{\left(j\right)}=\sqrt{\int_{0}^{\pi /\delta }\{(\Omega_{\delta }^{\left(j\right)}(K)-\Omega_{0}^{\left(j\right)}{\left(K)\right)}^{2}{\left(\Omega_{0}^{\left(j\right)}(K)\right)}^{-2}\}\;dK},\;j=1,2,$$

where *Ω*^(*j*)^_*δ*_(*K*) and *Ω*^(*j*)^_0_(*K*) are two dispersion curves (*j* = 1, 2) corresponding to the discrete and continuous model, respectively. By checking numerically, we have found that such a replacement is justified if the following two conditions are fulfilled simultaneously:

9.12 $$\varrho_{01}\delta^{4}\leq C_{1},\;\delta \leq C_{2}.$$

The first condition can be rewritten in the form of (7.1):

$$\Omega_{0}<\frac{\sqrt{C_{1}}}{a^{2}\sqrt{\varrho_{01}}}.$$

**Figure 17.**
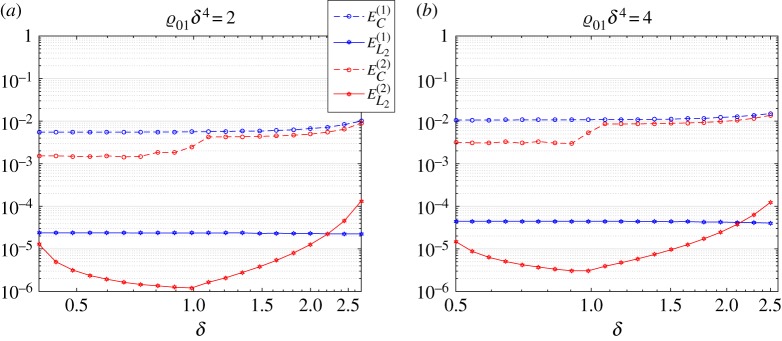
Deviations between the first two branches when the accurate formulation for the discretely distributed oscillators is replaced by the continuous variant. The continuous (homogenized) model has parameter choice (*a*) ϱ_01_*δ*^4^ = 2 and (*b*) ϱ_01_*δ*^4^ = 4 for different values of *δ* (horizontal axis) while on the vertical axis we show two different estimates in accordance with formulae (9.10) and (9.11). By blue (red) markers, we denote the first (second) branch of the respective dispersion diagram. (Online version in colour.)

Values for the constants *C*_1_ and *C*_2_ can be chosen from the estimates given in (9.10) and (9.11). We fit these results with the values *C*_1_ = 2 or *C*_1_ = 4 and *C*_2_ = 2.5. Those values are subjective and can be further discussed. As can be checked (see figure 17), they guarantee maximal deviation between the first two dispersion curves at the level of 1% in the point-wise sense (at the right-hand end of figure 17*a*,*b* (|*K*| ≈ *π*/*δ*)), while in the integral sense the accuracy is two orders higher.

## 10. Concluding remarks

This paper has presented a unified approach to the analytical description of the role of discrete and continuous dynamic microstructure embedded into an elastic body. Its generality enables the tackling of problems in both transient and time-harmonic regimes and the capture of exponentially localized waveforms in multi-scale elastic systems with embedded resonators. The use of lattice Green's functions leads to the modelling of waves in structured high-contrast waveguides with an emphasis on space and time non-locality.

This approach has worked effectively in finding analytical descriptions of wave by-pass systems (continuous, discrete and discrete–continuous), represented as a set of connected multi-scale resonators attached to the master solid. It has been demonstrated that, subject to appropriate tuning, the vibration in the master solid can be suppressed while the energy is transferred to the resonator wave by-pass.

This method has a wide range of applications in the modelling of earthquake-resistant elongated structures, such as long bridges or tall buildings. It also demonstrates analytical features of wave dispersion due to a coupling between the embedded resonator system and the master elastic solid for a variety of values of the stiffness and inertial parameters, which are essential in tuning of the by-pass systems for a given frequency regime.

The example illustrating dispersion of elastic waves in multi-scale systems, which include high-contrast wave by-pass systems, also shows the formation of stop bands and low-frequency standing waves. Finally, we have discussed the connection of the unified theoretical framework with the existing modelling results for structured waveguides.

## Data accessibility

This article does not contain any additional data.
